# Children's behavioural and emotional reactions towards living with congenital heart disease in Saudi Arabia: A grounded theory study

**DOI:** 10.1111/hex.13959

**Published:** 2024-02-27

**Authors:** Nada Dahlawi, Linda Milnes, Veronica Swallow

**Affiliations:** ^1^ Nursing Faculty King Abdulaziz University Jeddah Saudi Arabia; ^2^ School of Healthcare University of Leeds Leeds UK; ^3^ Department of Nursing and Midwifery Sheffield Hallam University Sheffield UK

**Keywords:** behaviour, children, congenital heart disease, emotion, grounded theory, stressors

## Abstract

**Background:**

A high incidence of children with congenital heart disease (CHD) was found in Saudi Arabia (SA). International literature reports that children with CHD exhibit behavioural and emotional issues due to experiencing hospitalisation and clinical treatments combined with a dearth of qualitative understanding of the experiences of younger children with CHD. Therefore, the aim was to explore the behaviour and emotions of 4–10‐year‐olds with CHD in SA through children's accounts of their own experiences and parental proxy reports of children's behaviour and emotions.

**Methods:**

Charmaz's constructivist grounded theory (GT) approach was used. Twenty single semi‐structured interviews of 10 child/parent dyads were undertaken at a hospital clinic in SA. Children's interviews were combined with an arts‐based approach using drawings, pictures and faces of emotions (emojis). Constant comparison analysis was undertaken. Consolidated Criteria for Reporting Qualitative Research guidelines was followed in reporting this study.

**Findings:**

A substantive GT: *children's behavioural and emotional reactions towards stressors related to living with CHD* was developed and provides new insights into children's and parents' perceptions of the children's behavioural and emotional reactions to living with CHD in SA. The theory proposes that children's reactions to living with CHD relate to medical treatment stressors, sociocultural stressors and physical change stressors. Several further factors influenced children's responses to these stressors.

**Conclusion:**

Children and parents in SA contributed to a new understanding of the relationship between CHD and children's behavioural and emotional reactions. In addition, findings support the need for early assessment of behaviour and emotions among children with CHD and the application of preventative and supportive measures for the children and their families in SA.

**Patient or Public Contribution:**

Before the research commenced, the developmental appropriateness of the proposed arts‐based data collection tools was tested with three healthy children aged 6–9 years old; the tools were then revised accordingly before the interviews were undertaken.

## 1 INTRODUCTION

Cardiovascular diseases, including congenital heart disease (CHD), were identified as a global burden, with an estimate of 17.8 million deaths globally in 2017.^1^ The Global Burden of Disease Study demonstrated that the global rate of CHD had declined by 34.5% (39.7% per 100,000 infants), and the global survival rate of children living with CHD had increased by 18.7% in 2017.^2^ Although this shows improvement, survival rates remains low and CHD is still a global concern.^2^

In Saudi Arabia (SA), a high prevalence rate of 14.8 CHD cases per 1000 live births was found from 2010 to 2013.^3^ Moreover, the Center for Disease Control and Prevention (CDC) identified CHD as one of the top 10 causes of death in children and young adults in SA.^4^ The high prevalence rate of CHD among children in SA indicates the significance of drawing attention to the need to improve the delivery of care to children with CHD who survive into adulthood.

To define CHD, it is described as defects in the heart that are present at birth and affect its natural function.^5^ With advances in management and treatment procedures, recent studies in children with CHD have focused more on improving their quality of life,^6^, ^7^ including exploring their psychosocial status.^8^, ^9^ These studies suggest a shift of focus towards improving psychosocial status, comprising behavioural and emotional outcomes of children with CHD.

Having any long‐term condition (LTC) is a risk factor for developing behavioural and emotional problems in children,^10^ for example, living with CHD. Among children with CHD, there is evidence of behavioural and emotional issues such as depression and social withdrawal.^11^ Moreover, the CDC^12^ reported that children with CHD, compared to healthy children, are prone to developmental, psychological and behavioural problems or impairments. In addition, a previous study in SA identified a need for future research on the psychosocial status of children with CHD.^13^ A recent review of the international literature focusing on children and young peoples' behavioural and emotional status as patients with CHD identified an important gap.^14^ While parents provided proxy reports on their children's behaviour and emotions in all studies and some studies reported perceptions of children over 7 years old, no studies explored the self‐perceptions of children under 7 years old on the impact of CHD on their behaviour and emotions or the factors that influence these.^14^

Hence, the aim of this study was to conduct in‐depth research using developmentally appropriate qualitative methods to:

1. Explore the personal views of children aged 4–10 years and their parents in SA on the children's behavioural and emotional reactions to stressors they face when living with CHD.
2. Determine the factors that influence children's reactions to the stress of having CHD.
3. Develop a theory to explain the behavioural and emotional reactions of children living with CHD.

## 2 METHODOLOGY AND METHODS

### 2.1 Design

This study followed the Consolidated Criteria for Reporting Qualitative Research guidelines.^15^ Charmaz's^16^ approach to grounded theory (GT) was used, as little is known about the perspective of young children living with CHD, including those in SA, or parents' views about these children's behavioural and emotional status.

Subjectivity is inevitable in data construction and Charmaz's.^16^ Ontological and epistemological assumptions matched those of the authors. The authors' constructivist ontological stance allowed for engaging personal experiences as child‐health nurses in interpreting, analysing and theorising the data. Therefore, Charmaz's constructivist ontological paradigm was most fitting to this study. Moreover, Charmaz's flexible approach advocates for multiple perspectives and complements the authors' view of parents and children as experts in children's support needs.

### 2.2 Participants and recruitment

Participants (*n* = 20) were recruited from cardiology clinics at a hospital in SA. Child participants (*n* = 10) were aged 4–10 years old and diagnosed with CHD; one parent of each child was also a study participant (*n* = 10). Purposive sampling was used for initial participant recruitment. Eligibility criteria for recruiting the initial sample are shown in Table 1. As concepts began to emerge during data analysis, theoretical sampling was undertaken. Theoretical sampling enables testing of the emergent concepts and theories from the data and allows for adding interview topics, recruiting new participants and/or changing study setting to collect further data.^17^ Participants' characteristics are shown in Table 2.

**Table 1:** Participants' eligibility criteria.

Criteria for initial sample (purposive sampling)	Inclusion	Exclusion
Children's age and gender	Male and female aged 4–10 years old.	Children younger than 4 and older than 10 years old.
Children's diagnosis	Diagnosed with CHD, who are pre‐ or postsurgery or cardiac catheterisation.	Diagnosed with: acquired heart diseases. syndromes or genetic disorders. physical or mental disabilities. psychological disorders.
Language	Arabic speakers: Saudi and non‐Saudi residents.	Non‐Arabic speakers, for example, Indian or Pakistani.
Parents	One parent of each eligible child.	
Criteria for theoretical sampling		
The interview questions were modified according to the analysis of the initially collected data. Thus, further participants had the opportunity to elaborate more about certain aspects that were generated from the analysis of the initial data. Further participants were approached for recruitment based on the initial data analysis (more female children and fathers were approached).		

**Table 2:** Participants' characteristics.

Families (F)	F1	F2	F3	F4	F5	F6	F7	F8	F9	F10
Criteria										
Age in years										
4								√	√^(4 and half)^	
5							√			
6	√		√	√						
7										
8		√			√					
9										√^(according to dad)^
10						√				
Birth order	First	Second^twin^	Third	First	Second	First	Fourth^youngest^	First	Fourth^youngest^	First
Severity/diagnosis										
Mild–moderate (a cyanotic CHD)	VSD	VSD + ASD	–	–	–	LVOT	Pulmonary artery atresia	–	Cardiomyopathy (and left ventricular dilatation)	–
Severe (cyanotic CHD)	–	–	TOF	TOF	TOF	–	–	Co‐aorta, TGA, DILV	–	TOF
Age at the time of diagnosis	After birth	8–9 months	1 month	9 months	After birth	Postbirth	3 months	40 days	40 days	After birth
Intervention										
Surgery (post/pre)	Postsurgery	Postsurgery ×2	Postsurgery	Postsurgery ×2	Postsurgery	Monitoring (follow‐ups)	Monitoring (follow‐ups)	Postsurgery waiting Second surgery	Monitoring ‐on medications (follow‐ups)	Postsurgery
Cardiac catheter (post/pre)	–	–	–	Postcatheter ×3	Postcatheter ×2			–		–
Age at the time of treatment	2 years	9 months	2.5 months	2 years	>3 years			3 months		8 months
Gender										
Male	√	√	√			√		√	√	√
Female				√	√		√			
Parents										
Mother	√	√				√	√	√	√	
Father			√	√	√					√

Abbreviations: ASD, atrial septal defect; CHD, congenital heart disease; co‐aorta, coarctation of aorta; DILV, double inlet left ventricle; LVOT, left ventricular outflow tract; TGA, transposition of the great arteries; TOF, Tetralogy of Fallot; VSD, ventricular septal defect.

### 2.3 Data collection

Individual, semi‐structured, in‐depth, face‐to‐face interviews were conducted with all children (*n* = 10) and (*n* = 7) of their parents in a hospital classroom, while three parents were interviewed by telephone according to their preferences. The interview topics were based on the first author's experience as a child‐health nurse and the literature, for example, a commonly used checklist for assessing children's behaviour and emotions (child's behavioural checklist).^14^, ^18^ See Table 3. With participants' consent, interviews were conducted in the Arabic language, audio recorded on an encrypted digital recorder, transcribed verbatim and anonymised. Translation from Arabic to English was done with anonymity and confidentiality protected. To cross‐check the translation, part of an anonymised script was shared with and agreed by a colleague who is fluent in both Arabic and English languages. All interviews were conducted and recorded by the first author.

**Table 3:** Parents' interview topic guide.

Interviewee	Topic	Example
Parents	Warm‐up questions	− ‘Tell me about your child's heart condition?’ − ‘What do you know about CHD?’
	Child's behaviour and emotions at home	− ‘How would you describe your child's behaviour at home?’ − ‘How do you think your child feels about his/her condition?’
	Child's behaviour and emotions at school, school performance and socialisation	− ‘How does your child's teacher describe him/her at school?’ − ‘Would you describe your child as a social child? Why?’
	Child's support	− ‘What can you tell me about how your child is dealing with having CHD’?

Abbreviation: CHD, congenital heart disease.

**Figure 1:**
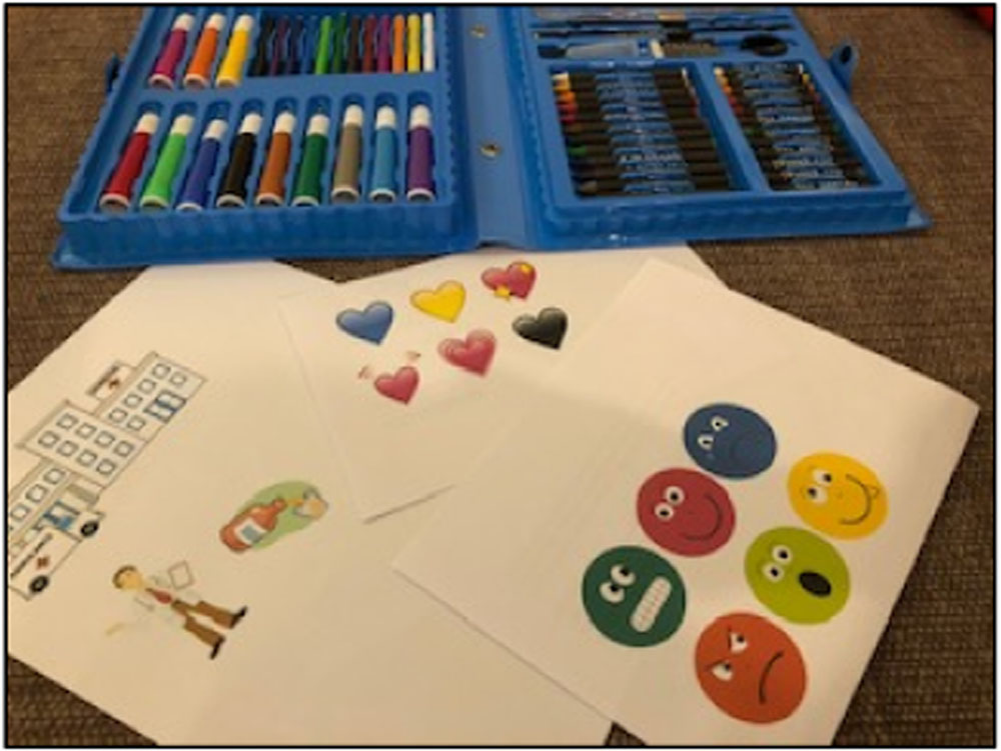
Arts‐based tools used in children's interviews.

Arts‐based approaches were used when interviewing children; these were appropriate methods for the children's developmental stages and enabled children's engagement in the interviews as they fitted with their daily life activities, skills and capacities.^19^ The arts‐based approaches worked effectively and efficiently with children who enjoyed drawing and using the pictures provided during their interviews. Also, asking each child to draw their face to convey what they feel about a particular topic was found to be effective among children in school settings.^20^ Arts‐based tools were first tested with (*n* = 3) healthy 6–9 years old and then revised; accordingly, the tools are shown in Figure 1. More information about the application of the arts‐based approach is provided in Table 4 and Supporting Information S1: File 1.

**Table 4:** Utilising arts‐based approach.

Arts‐based approach	Utilisations
Drawings	Children were invited to draw on a blank piece of paper and use their drawings to help them explain their feelings and behaviours. Hence, children's interviews were initiated with a drawing activity to attract the child's attention and engagement with the interview. Then, children were asked to draw themselves and to talk about what the child in the picture looks like or feels. Moreover, pictures of a hospital, doctors, nurses and hearts were used to help the child express feelings towards each picture.
Pictures of faces such as emojis	Pictures of emojis with emotional expressions of being happy, sad, crying or angry were also shown to the children, and they were asked to pick the face that best expresses their feelings towards different situations or places related to their CHD condition. For example, the children were asked how they felt about the pictures of the heart or hospital. Children usually pointed at the face that resembles their feelings, especially if they did not know how to express them in words.
Interpreting emojis and pictures of faces	Children interpreted the emojis and the other pictures according to what they meant to them. Certain faces and emojis are interpreted differently by some children. For example, the researcher/lead author asked a child about how getting tired easily made him feel. The child chose a sad face, but when the author asked what this face meant, the child perceived it as an angry face. Therefore, it was vital to ask the child each time they picked a face to check what exactly was the feeling they were expressing.
Autonomy in selecting a preferable arts‐based approach	While some children preferred to use pictures and emojis to describe themselves, others preferred only to draw. The researcher/lead author invited the children to choose and direct their preference in using the available tools of the arts‐based approach after introducing the tools to them. For the children who preferred drawing only, they liked to draw while talking throughout the interview.

Abbreviation: CHD, congenital heart disease.

### 2.4 Data analysis

In accordance with Charmaz,^16^ data collection was simultaneous with data analysis; this included coding, memo‐writing, constant comparison, theoretical sensitivity and categorising and theorising. Coding, which included three stages, initial, focused and theoretical, started following theoretical sampling and categorising. Theoretical saturation was determined to have been reached when no new concepts or categories emerged^16^; further data collection ceased at this point. At the categorising and theorising stage, the first author integrated her interpretation of the data based on personal experience and knowledge as a child‐health nurse. The analysis was undertaken manually by the first author using a Microsoft Word document. All authors participated in regular discussions about coding, categorising and theory generation.

To verify the rigour and accuracy of data collection, interpretations and reporting, guidelines provided by Charmaz^16^ on ensuring the quality of constructivist GT were followed, thus ensuring credibility, originality, resonance and usefulness^21^ (Table 5).

**Table 5:** Ensuring quality of constructivist GT.

Quality concept according to Charmaz	Example of how the quality concept was addressed in the study
Credibility	Writing memos, diagramming and peer debriefing by discussing the analysis process between the authors aided the researcher to maintain credibility.
Originality	The study demonstrates originality in that it provides fresh knowledge about the relationships between CHD‐related stressors and children's behaviour and emotions in Saudi Arabia.
Resonance	This study ensured that the emergent theory matched the description of the participants through peer debriefing between the authors and cross‐checking the interpretations and analytical thoughts with participants' interview's quotations.
Usefulness	The researcher ensured that the analysis and findings of this study generated a theory that can aid healthcare providers and families of children with CHD to understand the behaviour and emotions of children with CHD in Saudi Arabia and, thus, improve the care provision.

Abbreviations: CHD, congenital heart disease; GT, grounded theory.

### 2.5 Ethical considerations

This research was approved by the School of Healthcare Research Ethics Committee at The University of Leeds, as well as the Ethical Review Committee of the participating hospital in SA. Written and verbal information about the research, using an age‐appropriate and developmentally appropriate format, was provided to children and their parents. Children provided assent and parents provided consent for themselves and their children's participation.

Quotations from children and parents are referred to using the symbol ‘F’ with the family's study number, the child's age and gender. To protect confidentiality, pseudonyms were used for child respondents (see Table 2).

## 3 FINDINGS

### 3.1 The substantive theory

**Figure 2:**
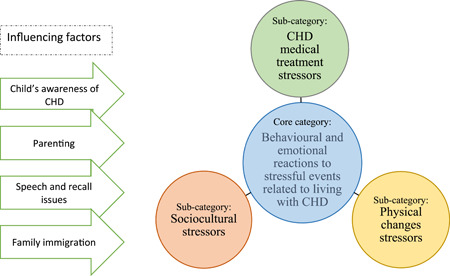
Relationships between core category, subcategories and influencing factors. CHD, congenital heart disease.

**Figure 3:**
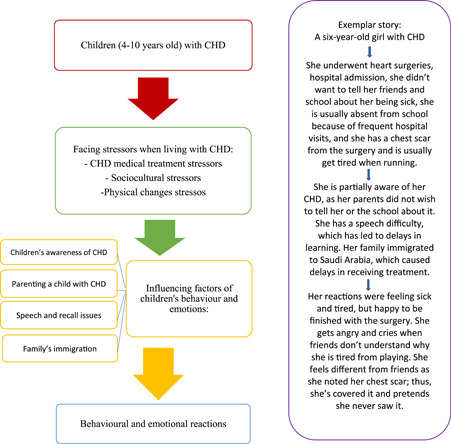
Flow chart of the proposed substantive theory. CHD, congenital heart disease.

**Figure 4:**
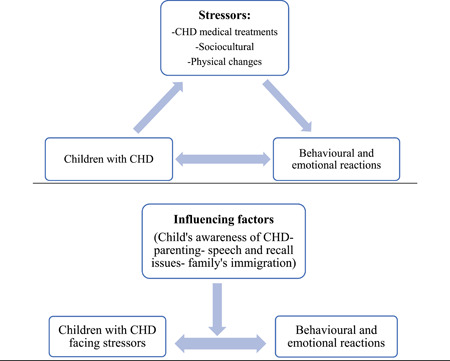
The relationships in the substantive theory with exemplar vignette. CHD, congenital heart disease.

The substantive theory *children's behavioural and emotional reactions towards stressors related to living with CHD was developed from the data*. The theory proposes that as children live with CHD, they face different *stressors*. These stressors are identified as subcategories: *CHD medical treatment, sociocultural and physical changes* which led to the children's behavioural and emotional reactions (see Figure 3). *Children's reactions to living with CHD* is the core category in this theory. Furthermore, several factors were found to influence children's responses to these three stressors *children's awareness, parenting, speech and recall issues*, and *family immigrations* as an overarching subcategory (see Figures 2, 3, 4 and Table 6).

**Table:** 

*Exemplar vignette from participants about the CHD medical treatments stressor*: A 10‐year‐old boy was diagnosed with CHD immediately after birth. He had been regularly followed up by doctors since birth and recently was scheduled to undergo heart surgery. Since then, he behaved excitedly about it and told his friends about the operation. However, when the researcher asked him what he felt about going into surgery, he reported his feeling of not wanting the surgery to be done. The child faced the stress of going into a surgical operation, and this stress mediated his behavioural and emotional reactions. Although he seemed excited by telling his friends about the operation's news, he actually felt that he did not want the surgery to be done.

**Table:** 

*Exemplar vignette from participants about children's awareness of CHD and parenting influencing children's physical changes*: A 6‐year‐old boy. He reported himself being scared of getting hurt when playing with friends. This was also reported by his mother, as well as his crying easily. Moreover, his mother reported telling her son to cover his chest scar and not tell his friends about his condition to avoid being hurt by his friends. The child demonstrated a lack of awareness of the condition in his interview as his mother did not want him to know about the CHD and hurt his feelings. The child's reactions to physical activity limitation and the presence of a chest scar were influenced by his awareness and parenting through the behaviour of hiding and covering the condition and the chest scar. He also reported feeling fearful of getting hurt when playing with friends, which can relate to his mother warning him to hide his scar to avoid getting hurt.

**Table 6:** Core‐category, subcategories and influencing factors.

Core category	Behavioural and emotional reactions to stressful life events related to living with CHD
Subcategories	Children showed different behavioural and emotional changes in response to stressful events that were related to the CHD condition, which were interpreted as stressors and considered subcategories in this theory. Stressors are identified as the factors which a person can face from any life changes, including possible challenges, burden, harm or pressure.^22^ For example, managing stress when dealing with the treatments and hospitalisations related to CHD, pressure from family and social interactions and physical changes associated with CHD.
Subcategory 1	CHD medical treatment stressors CHD correcting procedures. Hospital admissions and visits.
Subcategory 2	Sociocultural stressors Family relationships Schooling Friendships Sharing the news with others
Subcategory 3	Physical changes stressors Physical activity limitation Presence of scar from heart surgery
Factors influencing as an overarching subcategory	Several factors were found to influence children's reactions to the stressors (influencing the subcategories): *Children's awareness of CHD* influenced the way the children responded and reacted to the identified stressors. *Parenting* played an important role in children's awareness of CHD and their behavioural and emotional reactions towards all three stressors. *Children's speech and recall issues* contributed to the way they faced the stressors *Family's immigration* influenced children's experience with CHD treatment and the related stressors and thus they experienced behavioural and emotional reactions.

Abbreviation: CHD, congenital heart disease.

### 3.2 Subcategory: CHD medical treatment stressors

**Figure 5:**
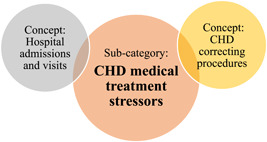
Concepts of subcategory: CHD medical treatment stressors. CHD, congenital heart disease.

This subcategory explains how children's CHD treatments, the different severities and types of CHD management procedures, hospital admissions or hospital visits act as stressors to children with CHD and could lead to their emotional and behavioural reactions (see Figure 5).

#### 3.2.1 CHD correction procedures

Before receiving treatments, children described themselves as being ‘Sad’ and ‘Scared and tired’, or ‘Sad and scared’ (F2–8 years old boy) that they felt before undergoing the surgeries, as opposed to them being ‘not scared and not sad or anything’, and ‘A happy boy’ (F2–8 years old boy) after the surgeries.

A girl who had undergone surgical and cardiac catheterisation management after the age of 3 years old described her immediate postoperative feelings as:

I was feeling tired and sick and I went to the bed and I slept … and they said I have to take a medicine. (F5–8 years old girl).

She also chose a pink heart and a happy face to describe her feelings and how her heart looked before the surgery (see Picture 1, 6).

**Picture 1:**
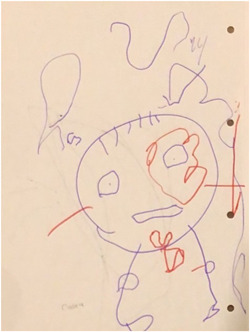
Child's drawing (F5–8 years old girl).

A father described his daughter as getting angry and easily crying before the surgery, which started to improve after the second surgery.

Before the first operation, she was very young, and things were not obvious.. she just used to be very angry and sometimes she would wake up at night and cry all the night and we couldn't sleep. (F4—father)

…she started to change, and she started to behave more.. but mostly she started to change after this surgery [second surgery]. (F4—father)

#### 3.2.2 Hospital admission and visits

Some children reported not being scared or distressed about hospital visits even after they experienced surgeries and frequent hospital admissions.

I don't get upset with the hospital. (F4–6 years old girl)

For some children, seeing their surgeon made them happy to visit the hospital.

I would be a little happy … Because I met the doctor…. (F6–10 years old boy)

However, staying in the hospital for several days was a concern for this child,

I don't want an operation … Who wants to sit on a bed for six days? (F6–10 years old boy)

Parents also reported that their children enjoyed the days of hospital visits and that they were only scared of needles or injections.

He doesn't get scared.. he is happy when he goes there [to the hospital].. he only gets scared of injections. (F2—mother)

Another parent reported that their child's attitudes towards hospital visits changed over time.

It is a matter of getting used to it.. after the first surgery she got used to it. (F5—father)

### 3.3 Subcategory—sSociocultural stressors

**Figure 6:**
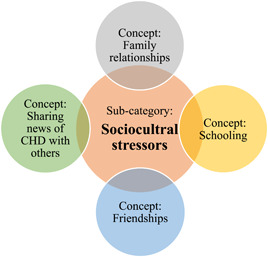
Concepts of subcategory: Sociocultural stressors. CHD, CHD, congenital heart disease.

This subcategory presents children's emotional and behavioural reactions towards the stress of dealing with social interactions and relationships while living with their CHD. Arab cultural aspects are interrelated with the social interactions of children (see Figure 1, 6). For example, parents' and children's unwillingness to share news of their CHD with others can be influenced by the Arab cultural beliefs of hiding private information from others.

#### 3.3.1 Family relationships

Relationships with siblings were reported to involve jealousy, perceptions of preferential treatments and competition.

They [brother and sister] upset me and fight with me.. they don't play with me…. (F3–6 years old boy)

This child drew his family members sitting together and playing on the left side of the picture and himself inside the house on the right side (see Picture 2).

**Picture 2:**
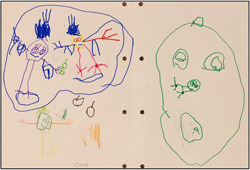
Child's drawing (F6 years old boy).

On the other hand, other children reported loving their siblings and related the feeling of love with spending time together in play (see Picture 3).

I love my sisters.. with my heart … They are my sisters they play with me every day. (F5–8 years old girl)

**Picture 3:**
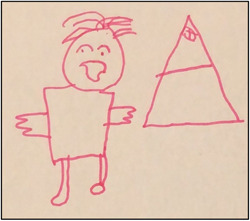
Child's drawing (F5–8 years old girl).

A mother described how much her son (with CHD) felt jealous of his sister, even though he received much more attention from his parents than his sister did.

He gets jealous very much … He says (everything is for her [sister] everything is for her) but she doesn't get the half of what he gets.. yes he gets jealous. He says, (no don't let her sleep next to you) he is not satisfied with the things he has…. (F6—mother)

However, despite feeling of jealousy, most participating children with CHD were reported by their parents as caring and kind towards their siblings or parents. For example,

He is caring … When I get sick or anything you will find him the only one who cries for me when he sees me. (F2—mother)

#### 3.3.2 Schooling

Behaviours of children with CHD at school were only reported by their parents. A mother reported her child's positive behaviour at school compared to their behaviour at home:

He [her son] is very quiet [at school] … I mean he is different at home. (F1—mother)

Whereas, another mother reported that her daughter's school complained of her daughter's frequent sleeping at school:

..it's only about the sleeping sometimes.. she goes to school and wants to sleep. (F7—mother)

Some parents pushed their children to do better at school, for example, a father said:

Sometimes when she fails [at school] I get angry with her … I think her memory is a little bit weak … My doubt is that it is maybe because of her condition. (F5—father)

#### 3.3.3 Friendship

Some children showed interest in being friends with other children who have CHD at the hospital:

Maybe …. Because if we [him and children with CHD] get tired, all of us will get tired together.. one tiredness [laughed]. (F6–10 years old boy)

On the other hand, another child did not want to look for friends with CHD because no one had the same heart operation as him:

No, he [the doctor] has no one like this. (F1 years old boy)

Parents reported their children getting special treatment from friends because they were aware of the heart condition; thus, they treated them gently.

They [her friends] know that she is sick, so they become soft with her. (F4—father)

Being angry and hitting between children with CHD and their friends was reported by children and parents.

They [his friend] shout at me.. and hurt me… I hurt them. (F8–4 years old boy)

One mother reported her son's excessive crying and his sensitive nature and stuttering (speech issue) whenever they made fun of him.

He is.. like I said.. he is sensitive.. for example, if the boys are just talking and discussing he would cry.. if they said things about him.. he would cry, and he hit them.. like light hitting. (F6–10 years old boy)

#### 3.3.4 Sharing news of their CHD with others

The children were selective in telling others about their conditions. For example, a child felt it was fine to tell only some of his friends.

Some of them know … They hear other teachers talking to each other and they hear them talking [about the condition]. (F2–8 years old boy)

In addition, another child talked about his condition and hospital visits only to family members and never to his friends who also had CHD.

Why should I tell them?.. actually two of them [the relative friends] they have the same as I got. (F6–10 years old boy)

Parents also reported some hesitancy.

Well.. it was between my husband and me.. even my family didn't know about it, only recently they knew.. I don't like to tell things like this.. you know! (F6—mother)

### 3.4 Subcategory: Physical changes stressors

**Figure 7:**
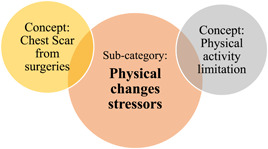
Concepts of subcategory: Physical changes stressors.

This section is about children facing the stress of having physical symptoms, for example, easily getting tired when playing, and physical changes, for example, a chest scar (see Figure 7).

#### 3.4.1 Physical activity limitation

Getting tired while playing can act as a stress for young children with CHD, who loved playing and being active. They also compared themselves with their healthy peers in terms of who gets tired easily.

….I sometimes get too much tired, and sometimes I get a little tired.. like this [pointing at face with tears].. my tears would be fallen like this … It [his heart] it would beat.. and if I set down I would become like this [pointing at a happy face]. (F10–9 years old boy)

Parents, as well, reported their children being physically active, yet easily getting tired.

I mean playing.. jumping.. walking up and down.. he gets tired.. he plays football, he likes football.. he likes to be active, but I have noticed something.. that when he is making a big effort, his lips turn green.. I mean around his mouth. So, they [at the hospital] have told me it's normal. (F9—mother)

A father compared her daughter's activity to that of her sister after she had undergone her first surgical operation:

She can play but not like her younger sister. (F5—father)

#### 3.4.2 Chest scar from heart surgery

The postoperative scar was identified as a stressor which interrupted physical self‐image and affected the way they felt and behaved towards their physical changes.

One child said

So, from here until here [showing the scar on his chest] and from my back and from here … No, not upset. I was happy when they did that [the surgery]. (F3–6 years old boy)

Parents and children chose to either talk or not talk about the presence of the scar with others

We [she and her husband] are trying not to talk about it [CHD]. I cover the scar.. I don't want the other kids to see it. I don't want him [her son] to concentrate on it … They would know his weakness point. I don't want him to get sensitive about it or be aware of it.. I don't want him to get inside the (picture of a sick child). (F1—mother)

Indeed, her child reported that he did not see his scar even though he showed his awareness of the scar's presence in other occasions during the interview.

Another father said:

…once, he [his son] was talking to me (Why I am like that dad?).. (Why did they do this on my chest?).. (My brother has nothing like this)… I told him when you were younger something happened and the doctor said that he had to check your heart.. but you are just like your brother. (F10—father)

### 3.5 Overarching subcategory: Influencing factors

#### 3.5.1 Children's awareness of their heart condition

Eight of the children became aware of their CHD either through their parents discussing the condition with them or because of frequent hospital visits.

Some children, who demonstrated awareness of their condition, described their happy feelings about having CHD, while other children reported negative feelings:

Angry … sad.. and I want to scream. (F8–4 years old boy)

For other children, even though they were aware of their conditions, they lacked some details of their journey with CHD management. They would only go to the hospital whenever their parents told them to.

Because Dad told me to. (F9–4 years old boy)

Even though some parents told their children about their conditions, they later regretted it.

I didn't wish to tell her.. I didn't want to talk about it … She might get scared … If someone would tell someone that you have got this and that, the person will be broken.. even if it's a child.. they can feel it. (F7—mother)

Moreover, parents chose to discuss or hide the condition from their children based on their own beliefs although parents' attitudes towards this varied considerably.

He has no idea about what he has got … when he gets older [will understand], but now at this age! I think no … they don't understand… (F2—mother)

#### 3.5.2 Parenting a child with CHD

Some children got special treatment from their parents because of their conditions, whereas other children experienced firm parenting styles.

A mother justified the special treatment as she felt sorry for him because her other children were ‘normal’.

Because you know thank god.. all of my other children are normal.. so I feel.. I mercy him … for example in the fridge there was only one chocolate. So, I would say [to his sister].. I know it's yours but you are older and you can understand so give it to him and I will ask your father to bring you some. (F9—mother)

This mother also reported incidents of jealousy between her boy with CHD and his siblings:

Everything will be for him first then the others. So, she [his sister] gets jealous because of this point. (F9—mother)

On other occasions, children themselves asked for preferential treatment from their parents.

She says, (Mom I am sick.. I have had an operation).. and if something is missing at home she says, (Dad where is the milk? The doctor said I should drink milk). (F4—father)

I feel he wants attention … especially if I am playing with his sister he would call me, (I want to talk to you). (F8—mother)

When it comes to disciplining their children with CHD, parents, particularly mothers, usually felt guilty about scolding them.

I spank him and then I pity him, and I would feel that I made a mistake by spanking him. (F2—mother)

#### 3.5.3 Children's speech and recall issues or difficulties

Children's speech or recall issues were found to influence their schooling and communication with their families and others. During the interviews, some speech issues were noted, for example, stuttering, pronunciation issues, saying unclear words or being unable to make clear complete sentences compared to their developmental stages.

A parent made a connection between his daughter's speech issue and her CHD:

Her speech was totally different.. you can't understand what she is saying, but after the surgery, she started to be fluent … once she got out of the hospital, she started to talk, and even her voice changed. (F4—father)

A speech issue affecting school performance was reported by a father,

She should be in grade three but we made her re‐do grade two. The alphabets, she can read them correctly but can't read a complete word. (F5—father).

Furthermore, recall issues or difficulties were reported for other children with CHD by their parents, which impacted their school performance:

She has a difficulty in recalling things. I feel that she can't memorise things quickly.. and she forgets things easily… (F7—mother)

#### 3.5.4 Family immigration

Two families immigrated from their home countries to pursue treatments for their children with CHD in SA, either because of a war in their home country or to seek specialist medical care. Accordingly, delays in diagnosis or in receiving treatments for the children occurred. Moreover, these children were noted to have speech and recall issues, which can be related to the delayed treatment and surgical corrections.

A father described being powerless.

..but then the war was started, and that's it. We couldn't do anything. Everything was closed and embassies and everything was closed over there.. it's a war.. and she stayed like this for a while … she became two years old when we got here [in SA]. (F4—father)

## 4 DISCUSSION

This is the first study to obtain the voices of 4–10‐year‐olds in SA with CHD and their parents; a substantive theory *children's behavioural and emotional reactions towards stressors related to living with CHD was generated*.

Integration of the emergent theory with existing theories

Some existing theories and models display similarities or differences to the current substantive theory. The biopsychosocial model Engel^23^ reports that biological, psychological and social factors influence individuals' experiences.^24^ Whereas, the current theory proposes that the psychological (behavioural and emotional) are reactions resulting from coping with stressors that include biological and social domains. Furthermore, the current theory adds the cultural aspect as a stressor for children with CHD. Moreover, the theories by Watson^25^ of behaviourism, operant conditioning reported by Skinner,^26^ and the James‐Lange theory and the Cannon‐Brad theory^27^, ^28^ share the same concept with the current theory, that is, that behaviour responds to stimuli, and emotions are reactions to events or stimuli.^27^, ^28^ The current theory, however, adds that other factors can influence how the children respond to those stimuli and reactions.

Contextualising stressors and influences on behavioural and emotional reactions in children with CHD in SA

Stressors found in the current study and associated with children's behavioural and emotional reactions are consistent with those reported in a recent meta‐analysis by Pinquart^29^ where children with severe LTCs who received intensive treatments showed high levels of posttraumatic stress symptoms.

Feelings of being scared, sad, sick and tired before invasive procedures that subside postintervention have been identified in the current study, and these also have been featured in previous literature. Preoperative behavioural problems were reported in a previous review on young children with CHD aged 2–3 years old, especially in severe cases, where there was a need for complicated surgical correction procedures at an early age.^30^ This was also demonstrated in another study^31^ where 5–15‐year‐olds with a range of CHD severities had lower stress and anxiety levels postsurgery than they had presurgery.

Even after undergoing surgery, children in this current study reported that they loved visiting the hospital and seeing the doctors who performed their operation. These findings are consistent with those of a previous study, which included children with acute and chronic illnesses aged 7–17 years old who had experienced more than 1 day of hospitalisation,^32^ reporting that hospital was a place where they go to be cured, and considering their doctors as friendly, kind and smart.

The chest scar from the heart surgery was a particular source of stress for children who questioned why they had these scars. This is in accordance with another study of children with the birth defect of oesophageal atresia, who were stressed by surgical scar; this led them to adopt coping strategies, for example, avoidance or acceptance of disease‐related body changes.^33^ Korean children and young adults with CHD felt hurt by others' curiosity about their chest scars when they were undressed in traditional bathhouses; in turn, as a coping strategy, they wanted to avoid the bathhouses.^34^

Children in this study enjoyed being physically active, even when they experienced tiredness and breathlessness. This finding corroborates the findings in a previous study,^35^ in which children with CHD felt encouraged to engage in physical activities, this enhanced their feelings of belonging with their friends.^36^

A previous study^37^ found that poor school adjustment among children with CHD was related to not telling their friends about CHD. These children demonstrated challenges when adjusting to school as they reached high schools and were not disclosing their conditions with others; thus, they were unable to develop close friendships.^37^

It was notable in the current study that if children were aware of their condition, they had the chance to decide whether or not they wanted to share the news of their CHD with others and plan their social interactions accordingly. This was also identified in previous research where young children with other LTCs found telling others about their conditions a stressful situation^33^, ^38^; but this is believed to be the first time this stressor was reported in children with CHD.

However, Arab families and healthcare providers do not involve children in discussions about their health issues believing children are less able to comprehend. Nevertheless, in the United Kingdom, children's understanding of their health condition demonstrated an understanding of health‐related information, as images and simple language were used when explaining the condition to children.^39^ Furthermore, children's anxiety and uncertainty before procedures were effectively managed when they understood the procedures.^39^

Moreover, the Arab culture appreciates privacy and keeping private news, such as sickness, from others which corresponds with a previous study conducted in SA,^13^ which found that the social and emotional stress were significantly higher in families of children with CHD, especially for those who underwent surgery.

Moreover, some parents treated their children with CHD more preferentially than siblings because they felt sorry for their children and worried that their children's CHD would deteriorate. This finding corresponds with accounts from a previous review,^40^ indicating that jealousy was reported by siblings of children with LTCs because of the special treatment the parents granted to their children with illnesses.^40^ It is vital to acknowledge that parenting a child with LTCs can also be stressful for parents.^41^

Immigration and speech and recall issues were influencing factors to the immigrant families of children with CHD. The struggles of immigrant families were extended due to poor socialisation, including school performance among immigrant children in the United States.^42^ Schooling of many migrant children worldwide is delayed or disturbed because of the migration and travelling process.^43^ These findings agree with those observed in the current study, which showed that school admission was delayed for immigrant children either because of the immigration process or their speech and recall issues. These findings also matched those of previous studies; a review by McKay^44^ demonstrates that immigrant children who require medical care and treatment have struggled to access healthcare facilities and encountered strains in adapting to the host country's healthcare system.^44^, ^45^

### 4.1 Implications and recommendations

This study informs healthcare in SA about the behavioural and emotional changes among children with CHD to enhance the medical and nursing care delivery and implement the necessary assessments and preventions. Moreover, this study highlights the need for developing interventional programmes in SA that aim to reduce children's behavioural and emotional issues because of CHD‐related stressors. Through these programmes, healthcare providers and policy makers can advocate for children with CHD and prepare children preoperatively, explain the surgery to them and encourage them to ask questions using child‐friendly approaches (e.g., arts‐based approach). Moreover, teachers and social‐care workers could provide specialised support to assist children who experience periods of absenteeism because of their sickness to re‐engage in schoolwork and physical activities with peers and minimise stress related to learning and friendship.

Recommendations for future research include testing the newly generated theory in other countries/cultures; exploring healthcare providers' views in SA about the behaviour and emotions of children with CHD; and investigating children's and family's needs for support in SA.

### 4.2 Strengths and limitations

Gathering accounts of 4–10 years‐olds through arts‐based interviews was a strength but also challenging. A limitation is an unbalanced sample regarding gender; hence, future research ought to recruit a more balanced sample.

## 5 CONCLUSIONS

This study introduced a novel substantive theory that highlights that children's behavioural and emotional changes are reactions towards stressors related to having CHD and are influenced by influencing factors. This theory adds extended meanings or concepts to what was already explained by other theories.

## AUTHOR CONTRIBUTIONS

**Nada Dahlawi**: Conceptualisation; methodology; investigation; writing—original draft; formal analysis; writing—review and editing; validation; visualisation; data curation. **Linda Milnes**: Supervision; writing—review and editing; validation; visualisation; resources; formal analysis. **Veronica Swallow**: Supervision; resources; validation; visualisation; writing—review and editing; formal analysis.

## CONFLICT OF INTEREST STATEMENT

The authors declare no conflicts of interest.

## Supporting information

Supporting information.

## Data Availability

Research data are not shared.
