# Supplementary material for: Children's behavioural and emotional reactions towards living with congenital heart disease in Saudi Arabia: A grounded theory study
Source: Health Expect. 2024 Feb 27;27(2):e13959. doi: 10.1111/hex.13959 (PMC10897868; doi:10.1111/hex.13959)
Supplement: Supplementary file 1 — Supporting information. [file HEX-27-e13959-s001.docx]

Supplementary file-1:

Arts-Based approach used in children’s interviews:

Arts-Based approaches were used, when conducting face-to-face interviews with children in the current study, which are appropriate methods for the children’s developmental stage. Face-to-face interviews offer a great opportunity to let the child actively engage in the interview using participatory techniques during the interview [1]. In addition, Arts-Based approaches are usually used in interviewing children to help encourage them to talk and express themselves. Arts-Based approaches are identified as child-oriented approaches as they fit with the children’s daily life activities, skills, and capacities [2]. One example is a study that involved children aged 4- 5-years old, who were hospitalised for minor procedures; they were effectively engaged during the interviews through using drawings, which acted as an ice-breaker tool for the children to feel relaxed and talk naturally [3]. To guide the interviews, interview topic guides were used. The children’s interview topic guide was designed to be appropriate to their age and developmental stage (Table 1).

Even though the author intended to show the pictures of the heart to introduce talking about the CHD, not all children talked about their heart condition directly when selecting their heart pictures. Some introduced talking about their condition when asked about the things that concerned them, or what were the things that they do not like about themselves. For instance, the child who mentioned becoming easily tired said that this was something he did not like about himself, the researcher/lead author was then able to probe using questions like “why”, or “what is it about getting tired?”. Then, the child started to talk about the CHD and the discussion about the condition was initiated.

Some children were more involved in free drawing than talking. In such cases, the researcher/lead author suggested drawing for a few seconds, then to stop and talk. Also, the researcher/lead author directed the children’s attention towards drawing things related to the interview’s points of discussion. For example, drawing their house, school, family members, friends, hearts, their doctors, or their hospital. The researcher/lead author then was able to talk to the child about related topics while letting the child use their drawing interest.

For all families, children and parents had the option for separate individual interviews to avoid any possibility of the child developing emotional harm or distress when parents were talking about their children and reporting children’s behavioural and emotional issues. The author obtained their informed assent or consent and revisited confidentiality and privacy considerations prior to the interviews. However, children and their parents were given the opportunity to make a choice about parents’ presence during the child’s interview. Half of the children (n=5) did not mind being interviewed without their parents present. The other children wished to be interviewed with their parent present.

Table 1: Interviews’ topic guide

| Participants | Interview Topic |
| --- | --- |
| Children | 1. Welcome the child, thank the child for participating, introduce myself.   Reassure that the parents have the choice to attend their child’s interview. |
|  | 1. Discuss ethics: assent, confidentiality limitations, and audio recording. Invite the child to ask questions. |
|  | 1. Warming up:   Getting the child to talk. Describe the play or the activities.   1. Do you want me to call you by your nick name? How old are you? 2. What is your favourite colour? 3. What is your favourite game? |
|  | 1. Feeling toward self and heart condition: 2. Can you draw yourself? Ask what this child looks like? Feels (introduce emojis or pictures of emotions to pick up from)? What is this child thinking about? 3. What are the things you like about yourself? What are the things you don’t like about yourself? Tell me more about yourself? Do you think you are smart? Beautiful? Why? 4. Can you draw your heart? What does your heart looks like? Why? 5. Can you draw yourself before and after being sick? How do you look like? 6. (Show the child pictures of hearts with different colours) Which of these is your heart? Why? Describe the heart you pick? How it is different than the other hearts? 7. (Show picture of hospital, medicine and doctors) Can you tell me what is building? What does this person (doctor) do? When you see this picture what do you feel (pictures of emotions)? 8. Can you draw me the hospital that you go to? Describe it? Do you like it? Why? 9. Can you draw your doctor? How does he look like? Do like your doctor? Why?   **Additions:**   1. **If you are going to discuss your condition/ or feeling with your friends, what would you tell them?** 2. **How did you feel before and after the heart surgery? (probing questions)** |
|  | 1. Self-perception towards their behaviour and emotion: 2. Can you draw your house? Who lives in this house? Do like your house? What do you usually do at home to spend time? 3. Can you draw your mother and father? Do love them? Why? Do you play with them? What do you love to do with them? Do you listen to what they tell you to do? Tell me more about it? What do you feel when they ask you to do things (pictures of emotions)? 4. Can you draw your siblings? Which one is your best? Why? Do you like to play together? Why? **(Additions): Do you get jealous of your siblings? How do you feel if parents or others are taking care of your siblings?** 5. Do you think your family loves you? Why? Does anything make you upset or sad at home? What is it? 6. What are the things make you happy? 7. What are things make you sad? 8. Do you cry a lot? Tell me more about it? 9. Is there anything you afraid of? Tell me more about it? 10. Do get angry all the time? What makes you angry? 11. Do you think you have special ideas or thoughts? Like what?   **Additions:**   1. **Tell me more about what you mean by being angry and crying (if the child mention them).** |
|  | 1. School activities and socialisation: 2. Do you go to school? What grade? 3. Can you draw your school? Do you like your school? Tell me about your school. How are your grades? 4. Do have friends at school? How many? Do you like them? Do they like you? Tell me more about it? 5. Do you feel that you are different than them? Tell me more about it? 6. Does anyone / anything bother you at school? Tell me more about it? 7. Do you think that people like you? Tell me more about it? 8. Do you like to stay alone or with people? Tell me more about it? 9. Are there any activities you are joining at school or outside school? Do like these activities? Tell me more about it? 10. Is there any place that you enjoy visiting? Why do you like this place?   **Additions:**   1. **Did you tell your friends or others about your condition? why?** 2. **Have you ever talked to other children who have same heart condition? Tell me more about it? How would you feel if you tell anyone? Tell me more about it?** 3. **Do you enjoy playing and running? Where do you play and with whom? How do you feel when playing? What do you do if feeling tired? How do you feel about being tired?** 4. **(if having speech or recall issues): How do you feel about having this difficulty? Tell me more.** |
|  | 1. Coping and support: 2. You told me that you have heart condition? how do you fell about it? Tell me more about it? 3. Do you think it will be useful to talk to doctors and nurses about the things that bother you?   **Additions:**   1. **Do the doctor talk to you about your condition?** 2. **Did your parents or other people explain the condition to you?** |
|  | 1. Closing and offering time for questions: 2. Thank you very much for talking to me 3. Is there anything else you want to tell me about before leaving? 4. Do you have any questions? |

References:

1. O'Reilly, M. and N. Dogra, *Interviewing children and young people for research*. 2016: Sage.

2. Carter, B. and K. Ford, *Researching children's health experiences: The place for participatory, child‐centered, arts‐based approaches.* Research in Nursing & Health, 2013. **36**(1): p. 95-107.

3. Swallow, V., J. Coad, and A. McFayden, *Involving children, young people and parents in knowledge generation in health and social care research*, in *McGraw Hill*. 2007.
